# New Approach for Preparing In Vitro Bioactive Scaffold Consisted of Ag-Doped Hydroxyapatite + Polyvinyltrimethoxysilane

**DOI:** 10.3390/polym13111695

**Published:** 2021-05-22

**Authors:** Marzieh Rabiei, Arvydas Palevicius, Reza Ebrahimi-Kahrizsangi, Sohrab Nasiri, Andrius Vilkauskas, Giedrius Janusas

**Affiliations:** 1 Faculty of Mechanical Engineering and Design, Kaunas University of Technology, LT-51424 Kaunas, Lithuania; arvydas.palevicius@ktu.lt (A.P.); sohrab.nasiri@ktu.edu (S.N.); andrius.vilkauskas@ktu.lt (A.V.); giedrius.janusas@ktu.lt (G.J.); 2 Advanced Materials Research Center, Department of Materials Engineering, Najafabad Branch, Islamic Azad University of Najafabad, Najafbad Isfahan P.O. Box 85141-43131, Iran; rezaebrahimi@iaun.ac.ir

**Keywords:** Ag-doped HA, mechanochemical process, spark plasma sintering, open porosities, simulated body fluid, bioactivity

## Abstract

Recently, researchers have focused on the biocompatibility and mechanical properties of highly porous structures of biomaterials products. Porous composites are a new category of bioengineering that possess excellent functional and structural properties. In this study, the physical and mechanical properties of prepared doped silver (Ag)-hydroxyapatite (HA) by the mechanochemical and spark plasma sintering (SPS) methods were investigated. The influence of dopant on phase formation, structural properties, mechanical properties and morphological characteristics was investigated. Furthermore, in this case, as a new approach to produce a porous scaffold with an average size of >100 µm, the hair band was used as a mold. According to the Monshi–Scherrer method, the crystal size of scaffold was calculated 38 ± 2 nm and this value was in the good agreement with average value from transmission electron microscopy (TEM) analysis. In addition, the stress–strain compression test of scaffold was considered, and the maximum value of compressive strength was recorded ~15.71 MPa. Taking into account the XRD, TEM, Fourier-transform infrared (FTIR), scanning electron microscope (SEM) and energy dispersive X-Ray analysis (EDAX) analysis, the prepared scaffold was bioactive and the effects of doped Ag-HA and the use of polyvinyltrimethoxysilane (PVTMS) as an additive were desirable. The results showed that the effect of thermal treatment on composed of Ag and HA were impressive while no change in transformation was observed at 850 °C. In addition, PVTMS plays an important role as an additive for preventing the decomposition and creating open-microporous in the scaffold that these porosities can be helpful for increasing bioactivity.

## 1. Introduction

Taking into account the existence of Calcium Phosphates (CaPs) in the body, researchers nowadays have considered CaPs for the replacement and repair of injured bones. One of the most desirable and well-known CaPs groups is associated with hydroxyapatite (HA) [1,2]. The chemical formula of hydroxyapatite is Ca_10_(PO_4_)_6_(OH)_2_ and it differs little from the bone tissue [3]. According to the knowledge of crystallographic structures, there are two types of HA structures: hexagonal and monoclinic. Mostly the hexagonal HA is suitable for the biomaterials industry, as the monoclinic HA is not more stable in bioactive properties [4]. In addition, one of the challenges for using HA as a bioactive component is related to the likelihood of infection; therefore, the use of antibacterial materials is the best way to solve this problem [5]. On the other hand, the studies have proven that silver (Ag) has antibacterial properties [6,7,8]. Moreover, one of the best elements for biocompatibility is Ag, since it is related to the high value of antibacterial coefficient (100%) [9,10]. However, the biocompatibility properties of Ag are directly dependent on the strength, density and the manufactured final phase of bioactive composites. There are several mechanisms that involve Ag in the interaction with biological macromolecules [11]. In addition, the Ag^+^ has been shown to bind to protein functional groups, leading to protein denaturation [12]. Polyvinyltrimethoxysilane (PVTMS) has a functional group like silanol (Si-O-H) which can help in bonding; therefore, it is useful for preventing the decomposition of composites [13]. PVTMS is a type of component-based on polysiloxane that is of particular interest due to its dense structure of siloxane cross-linked with polymeric groups, in addition, PVTMS is bioactive and enhances mechanical properties by providing a stable Si-O-Si framework [14]. Recently, the mechanochemical process has been considered as a new approach. In this process, the mixed powders are affected by the collisions between the high energy balls and the container, and this interaction continues until the bonding and fracture rate are balanced [15,16]. Overall, several parameters are effective in the mechanochemical process, such as the grinding rate [16], the grinding time [17,18], the weight ratio of balls to powder [17], the grinding atmosphere [17,19] and the applied temperature [20]. Another method for sintering conductive and non-conductive components is spark plasma sintering (SPS). The first studies on SPS date back to 1930 [21]. The general theory of SPS is attributed to the micro spark plasma. According to this theory, there is an electrical discharge and the instantaneous generation of a spark plasma with high temperature in a local small area between the powder particles in one second [22]. As mentioned above, the HA is very sensitive to temperature, and the variation of phases and decomposition resulting from the high temperature ~ 1100 °C of HA are the reason for the selection of SPS process for sintering [23]. In this study, the SPS process is very applicable by preventing the collapse and preventing the growing grains that these properties that are strictly effective for bioactivity and mechanical properties [24]. There are several studies that have been conducted by SPS to produce HA or composites composed of HA. For example, Shen et al. used the SPS process to fabricate transparent HA ceramics with nano-grain structure at high temperature [25]. In addition, Zhang et al. studied the effect of HA on microstructure and bio composites and the method of synthesis was SPS [26].

Simon et al. fabricated composites of bioactive glass/polymer and used poly-96L/4D lactide for strength [27]. In another study, Goller et al. focused on the process variables in electrospinning gelatin/silver nanoparticles/bioactive glass for tissue engineering applications [28]. However, in all the studies related to the use of silver as antibacterial part and polymer as strength part, the creation of porosity to increase biocompatibility and the use of hydrophobic polymers such as silane groups of polymers were overlooked and not discussed. To overcome the limitations of the size of porosity in the fabrication of composite scaffolds, utilizing the hair band was proposed as a new approach. The multi-pore scaffold with local pores was proposed to improve the in vitro bioactivity response of scaffolds. The aim of this study is to prepare scaffold of Ag-doped HA + PVTMS for the first time. In the present study, a mechanochemical process was carried out to synthesize Ag-doped HA. Taking into account the differential scanning calorimetry (DSC) curve of the soaked hair band with slurry, the final sintering temperature was chosen 350 °C and a porous scaffold was fabricated. The porosities were opened, which can be suitable to increase the ratio of biocompatibility. The doping mechanism, structural evolution, morphological properties, mechanical properties and bioactivity analysis were studied by spectroscopic and microscopic techniques. Frankly, the novelty of this work was the use of the hair band to create open porosity with the size of >100 µm, as well as the use of PVTMS to prevent the collapse of scaffold under heat treatment.

## 2. Experimental Methods

### 2.1. Materials and Instruments

In this study, calcium nitrate tetrahydrate (Ca(NO_3_)_2_·4H_2_O), phosphorus pentoxide (P_2_O_5_), silver nitrate (AgNO_3_) and *vinyltrimethoxysilane* (VTMS) (merck) were utilized as the precursors. Powders X-Ray, phase series were confirmed by X-ray diffraction (XRD) and performed on a Philips XRD diffractometer, Cukα radiation was used at 40 KV, 30 mA, step size of 0.05° (2ϴ) nd scan rate of 1°/min (AXS GmbH, Kaunas, Lithuania). Furthermore, X’Pert software was used for qualitative analysis and report of width diffraction peaks (rad, β) at full width half maximum (*FWHM*) in different 2θ values according to the situation of peaks (Version 4.9.0). In addition, differential scanning calorimetry (DSC) were carried out by STA-BAHR (Iran, Isfahan). Fourier-transform infrared spectroscopy (FTIR) spectra of the compounds was attached in the potassium bromide (KBr) powders and the instrument that was used was a Perkin-Elmer Spectrum BX FT-IR spectrometer (Iran, Isfahan). Moreover, transmission electron microscopy (TEM) Tecnai G2 F20 X–TWIN with acceleration voltage from 50 to 80 KV was utilized (Iran, Tehran). For chemical elements of components, an energy dispersive X-Ray (EDX) spectrometer Phillips/FEI 149 Quanta 200 was utilized (Iran, Tehran). In addition, scanning electron microscope analysis (SEM) Phillips/ FEI Quanta 200 was used to study the morphology of the compounds and scaffold (Iran, Tehran). In addition, simulated body fluid (SBF) was prepared according to the method of Kokubo et al. [29]. Considering the temperature at 37 °C, materials were added and dissolved according to the Kokubo method. Afterward, (CH_2_OH)_3_CNH_2_ and HCl (merck) were added (dropwise) to achieve a pH of 7.40 (final pH), then the temperature was decreased (20 °C) and distilled water was added [29]. In this study, the measurements of the samples were repeated three times and the statistical results were reported as the average of the values.

### 2.2. Synthesis of HA

**Figure 1:**
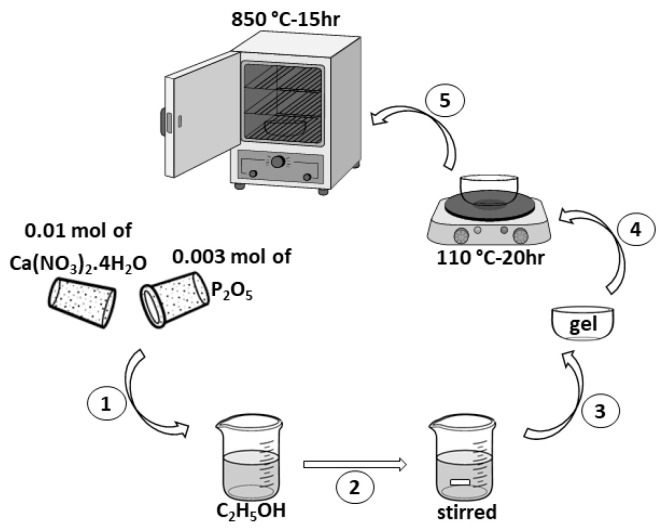
The schematic flow diagram of the synthesis route of HA.

**Figure 2:**
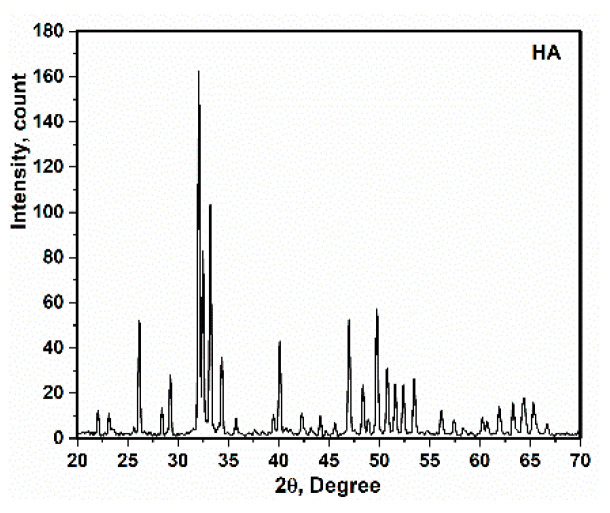
X-ray diffraction of HA.

According to Figure 1, hydroxyapatite (HA) was synthesized by sol–gel method. Taking into account the Ca/P ratio of HA, calcium nitrate tetrahydrate (Ca(NO_3_)_2_·4H_2_O) and phosphorus pentoxide (P_2_O_5_) were used in the molar ratio of 10:3. The following steps were performed: (1) Ca(NO_3_)_2_·4H_2_O) and P_2_O_5_ were dissolved in 10 mL of ethyl alcohol (C_2_H_5_OH) and distillated water. (2) The product was stirred at 350 rpm for 2 h. (3) The gel was prepared at the bottom of the dish. (4) The gel was then dried at 110 °C in air for 20 h. (5) Heat treatment at 850 °C for 15 h was considered for sintering. This is similar to the method is presented in [30,31]. The X-ray diffraction of synthesized HA is presented in Figure 2. 

### 2.3. Polymerization of Vinyltrimethoxysilane

**Figure 3:**
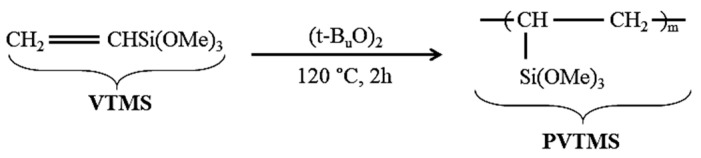
Synthesis route of PVTMS.

Vinyltrimethoxysilane (VTMS) was polymerized to 20-mers. In addition, PVTMS was caused to enhance the thermal stability and physical properties of scaffold. PVTMS was prepared with tertiary butyl peroxide as an initiator under reflux for 2 h at 150 °C in flowing nitrogen [32,33]. In addition, the synthesis route of PVTMS is shown in Figure 3.

### 2.4. Ag-Doped HA by Mechanochemical and Spark Plasma Sintering Process

**Figure 4:**
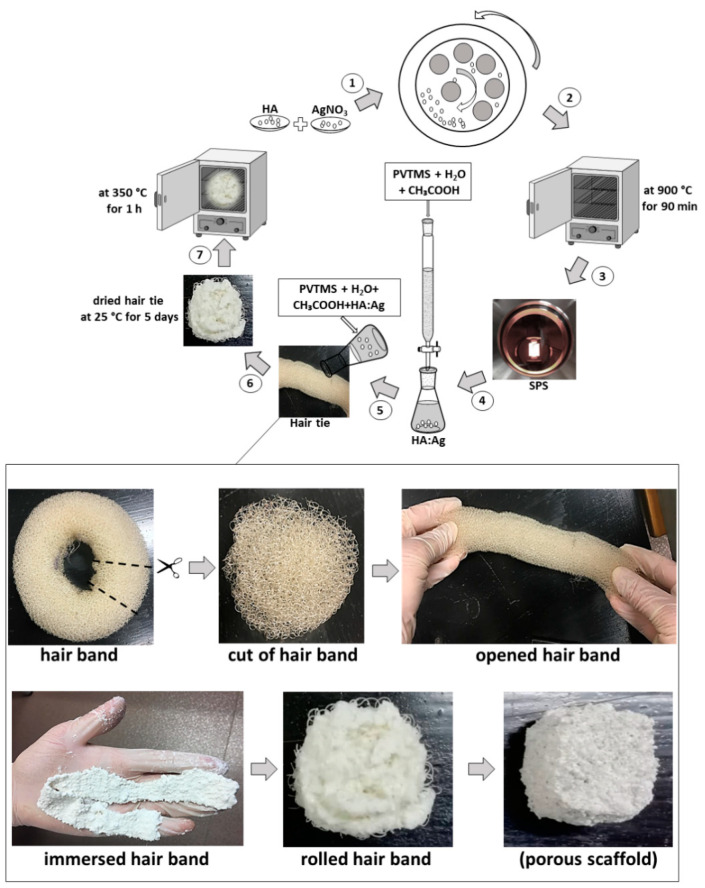
The routes of fabricated Ag-doped HA+PVTMS scaffold.

In this study, silver nitrate (AgNO_3_) with 99% purity was used as the dopant. The amount of replacement of Ca^2+^ ions by Ag^+^ corresponds to the formula ${\mathrm{Ca}}_{10-X}{\mathrm{Ag}}_{X}$(PO_4_)_6_${\left(\mathrm{OH}\right)}_{2-X}{\;}_{X}$. In this case, X = 2 and ${\;}_{X}$ is represented as the x-mole of OH-vacancy formed by doping X-mole of Ag in the HA lattice. Moreover, the ratio of (Ca + Ag)/P was set to 1.67, while the degree of cationic substitution was changed. According to Figure 4, (1) The powders of HA and AgNO_3_ were ground by using a high-energy planetary ball mill via a mechanochemical process at 4 h. In mechanochemical process, hard chromium steel shells and balls with a diameter of 20 mm were used. This process was carried out at ambient atmosphere and rate of 500 rpm with a ball to powder ratio of 15:1. In addition, to prevent agglomeration of the particles, the machine was stopped per 45 min. (2) After the end of mechanochemical process, calcination was carried out at 900 °C, at a heating rate of 10 °C per minute for degassing (90 min). (3) Taking into account the fact that HA is very sensitive to temperature, transformation and decomposition, the SPS process was considered. In this process, the powder was placed in the graphite mold (diameter = 20 mm), and pressure and temperature values were set 50 MPa and 600 °C for 10 min, respectively. The route of the fabricated Ag-doped HA composite is shown in Figure 4.

### 2.5. New Approach of Fabrication Ag-Doped HA+PVTMS Scaffold

(4) Component of a slurry consisted of Ag-doped HA (93 wt%)/PVTMS (5 wt%)/H_2_O (1 wt%)/CH_3_COOH (1 wt%) were prepared. (5) In addition, a hair band (commercial) was chosen as a new approach to obtain open porosities, (6) which was immersed in the slurry for 5 days at room temperature. According to Figure 4, the hair band was cut and opened and the tape was pulled out, the taped hair band was immersed in the slurry and then the tape was rolled up. (7) Then, the product was placed in the furnace at 350 °C for 1 h according to the DSC curve, vinyl groups and temperature of the burning hair band. Finally, the porous scaffold consisted of Ag-doped HA+PVTMS was fabricated. It is necessary to mention that HA is considered as a bioactive matrix due to the presence of calcium phosphate groups. Moreover, Ag is the best metal for an antibacterial environment. In addition, acetic acid (CH_3_COOH) has played an important role as a dispersant for particles, especially for Ag, to prevent the formation of colloidal particles and to prepare small particles. PVTMS is bioactive polymer, as well as PVTMS is an organo-silane molecule and provides a hydrophobic environment in the composite. Since water can easily diffuse into the polymer structure, and it can lead to the breakdown of intermolecular forces and create the voids in the polymer [34]. The effect of PVTMS is related to the implementation of the hydrophobic environment and the removal hydroxyl groups via silane groups in the PVTMS structure. Nevertheless, bonds of C and siloxane with hybrid sp^2^, sp^3^ are more stable and uniform, as well as, PVTMS is an intermediate for bonding and it will lead to improvement in mechanical properties and adhesion. Therefore, PVTMS prevents a hydrophilic ambient and the collapse of the scaffold [35]. 

PVTMS played an important role as a drying control chemical additive (DCCA) to prevent cracking and shrinkage of scaffold. As shown in Figure 3, the free radicals of PVTMS structure can contribute to the flexibility of the scaffold through the bonding between C-C and siloxane (Si-O-Si). Furthermore, PVTMS has carbon chains, and these chains can help to prevent the collapse of scaffold during the heat treatment, leaving solvent and volatile materials and creating porosity without damaging the scaffold.

## 3. Results and Discussion

### 3.1. DSC Analysis of Rolled Hair Band Consisted of Ag-Doped HA+PVTMS+CH_3_COOH+H_2_O

**Figure 5:**
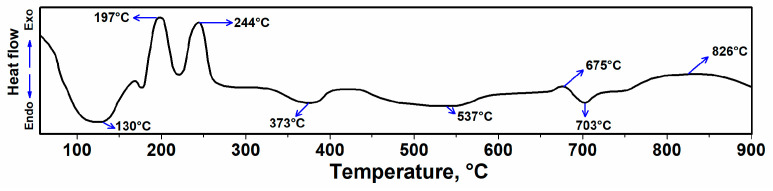
DSC of rolled hair band consisted of Ag-doped HA+PVTMS+CH_3_COOH+H_2_O.

The DSC of the rolled hair band consisted of Ag-doped+HA+PVTMS+CH_3_COOH +H_2_O is shown in Figure 5. The endothermic spectra at ~130 °C is related to the evaporation of physically absorbed water on the scaffold surface [36]. The exothermic peaks at 197 and 244 °C are related to the self-combustion process of the burning hair band with the evolution of volatile products [37]. Chemically absorbed water requires more energy to be released from the structure. The rapid decomposition of PVTMS occurs at 373 °C. Water in pores requires more heat to be released due to the capillary effect, so the final heat treatment temperature of 350 °C (<373 °C) was chosen with considering the collapse temperature. In addition, the peak at ~373 °C can be related to desorption of chemically bound absorbed lattice water, removal of O-H groups [30,38] and at 537 and 675 °C are due to dehydration and at 703 °C it is due to condensation of HPO_4_ and crystallization of HA and decarburization-phase transformation into HA such as beta-tricalcium phosphate (β-TCP) [39]. Accordingly, the exothermic temperature at ~832 °C can be attributed to the dehydroxylation of HA leading to the decomposition of HA to oxyhydroxyapatite [40,41]. According to Figure 5, the temperature to prevent degradation was chosen as 350 °C (for 1 h), and this temperature is an optimum value because at this temperature, the hair band is burned, and the porosities are created without damage and degradation to the scaffold. At high temperatures the composite is degraded, but we do not need high temperatures, because the body cannot tolerate more than normal temperature (> 39 °C); therefore, the thermal stability of 350 °C is perfectly suitable.

### 3.2. X-ray Diffraction and TEM Analysis

**Figure 6:**
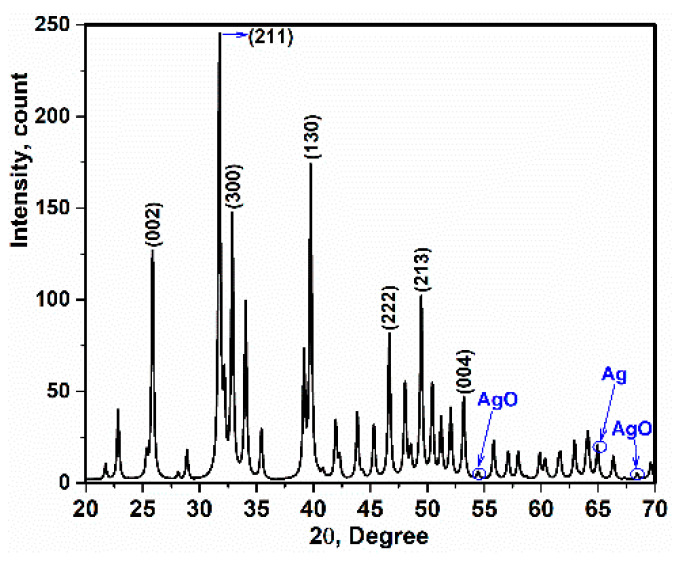
X-ray diffraction of Ag-doped HA+PVTMS scaffold.

X-ray diffraction of the Ag-doped HA+PVTMS scaffold is shown in Figure 6. It is clear that the pattern is close to the pure HA pattern [3,42]. The precursors such as Ca(NO_3_)_2_·4H_2_O), P_2_O_5_ and AgNO_3_ disappear, and single phase of Ag-doped HA+PVTMS is produced. In addition, the absence of specific peaks of components such as AgNO_3_ and Ag_3_PO_4_ and the agreement of the XRD pattern (Figure 2) with the XRD pattern of pure HA were performed. Further, Ag^+^ ions being replaced in the HA lattice are confirmed. Additionally, a slight shift of the peaks to low and high angles can be associated with the replacement of carbonate rather than phosphate groups. Moreover, the characteristic main peaks of (002) at 2θ = 25.86°, (211) at 31.86°, (300) at 32.89°, (130) 39.75°, (222) at 46.72°, (213) at 49.45° and (004) at 53.11° are investigated, showing that the cationic substitution not only prevents the formation of Ag-doped HA+PVTMS but also stabilizes at room temperature, as confirmed in [43]. Moreover, the peaks at 54.48°, 64.96° and 68.43° are attributed to the AgO and Ag tandemly [44]. Similarly, X-ray diffraction of Ag-doped HA is reported in Refs [45,46]. The lattice parameters of the HA and Ag-doped HA+PVTMS structures were recorded as (a = 9.53 Å, c = 6.76 Å) and (a = 9.59 Å, c = 6.86 Å), respectively. Therefore, the a and c parameters of the Ag-doped HA+PVTMS structure increased, which is due to the larger ionic radius of Ag compared to Ca and it is related to the proper bonding between Ag ions and the HA structure [47]. 

**Figure 7:**
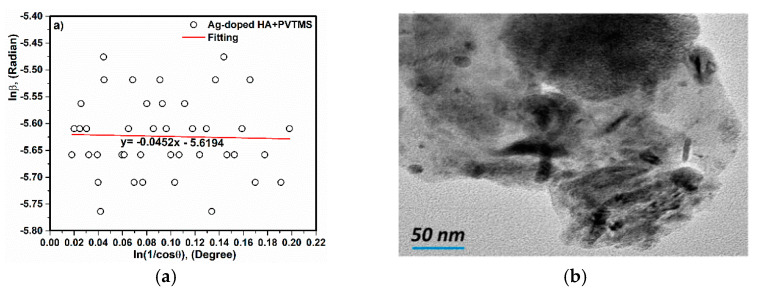
(**a**) Linear plots of the Monshi-Scherrer equation and (**b**) TEM image of scaffold.

According to the Monshi–Scherrer method, the nanocrystalline size of the Ag-doped HA+PVTMS scaffold was calculated. Furthermore, the linear plot of Ln β (β in radians) vs. Ln (1/(Cos ϴ)) (degree) can be a linear plot for all chosen peaks (Figure 7a). In equation 1, β is the full width at half maximum of the peak in radians, K is the shape factor, usually assumed to be 0.89 for ceramic materials, λ is the wavelength of the radiation in nanometers ($\lambda_{{\mathrm{Cu}}_{K\alpha }}$ = 0.15405 nm), ϴ is the diffraction angle of the peak and L is the nanocrystal size [3,48]. The value of the intercept is calculated as e^(−5.6194)^ = 0.0036, therefore $\frac{K\;\lambda }{L}$ = 0.0036, and the crystal size is calculated as L = 38 ± 2 nm. In addition, the grain size was carried out on TEM image and (D_TEM_) is ~ less than 50 nm (Figure 7b). For scaffold, the D_TEM_ value is almost larger than the extracted crystallite size from Monshi–Scherrer equation, which can be explained by the fact that a grain consists of more than one crystallite and it is related to the nucleation and growth of the particles [48,49,50,51].

(1) $$\mathrm{Ln}\;\beta =\mathrm{Ln}\;(\frac{K\lambda }{L})+\mathrm{Ln}\;(\frac{1}{\mathrm{Cos}\;\theta })$$

### 3.3. Fourier-Transform Infrared Spectroscopy (FTIR) Analysis

**Figure 8:**
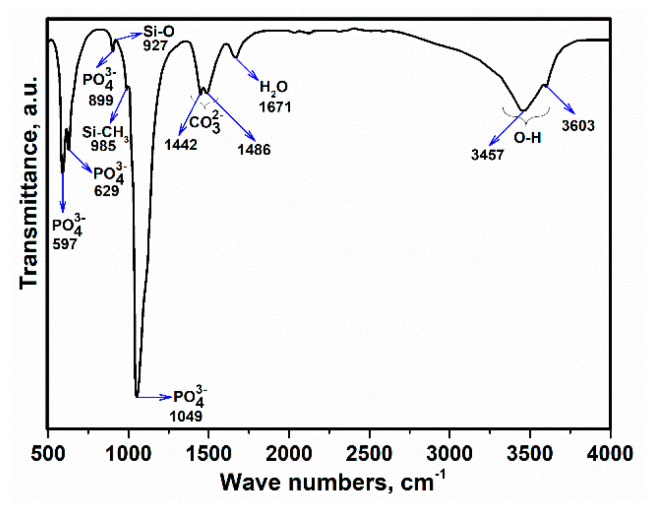
FTIR spectra of Ag-doped HA + PVTMS scaffold.

The FTIR spectrum of the Ag-doped HA+PVTMS scaffold is shown in Figure 8. This spectrum corresponds with FTIR spectra of HA [52,53]. Moreover, the strong stretching mode is associated with ${\mathrm{PO}}_{4}^{3-}$ at 1049 cm^−1^ and the broad spectrum is due to the bonding between O and H. The evidence of the existence of carbonate groups in HA corresponds to the wave numbers at 1442 and 1486 cm^−1^ (belonging to the –CH_3_ group) and all –CH_3_ groups have implied the existence of PVTMS. Moreover, the wave number values of 927 and 985 cm^−1^ are due to the effect of PVTMS [54]. Based on the study of Gibson et al. in the HA structure, the AB-type substitution of the phosphate by the carbonate can improve the bioactivity and mechanical properties as well as lowering the sintering temperature [55]. Therefore, according to the AgNO_3_ content in the fabrication process of scaffold, there is no impressive tangible difference between the FTIR of pure HA and the FTIR of Ag-doped HA+PVTMS, except the intensity and broadening of the spectrum in some regions, which can be attributed to the substitutions of cations and anions in the HA structure. Furthermore the existence of main bonds are corresponded with [8,10], overall, the absorption bands are characteristic bands for HA, which are the same as previously reported in [56].

### 3.4. Study of Morphology by SEM Analysis

**Figure 9:**
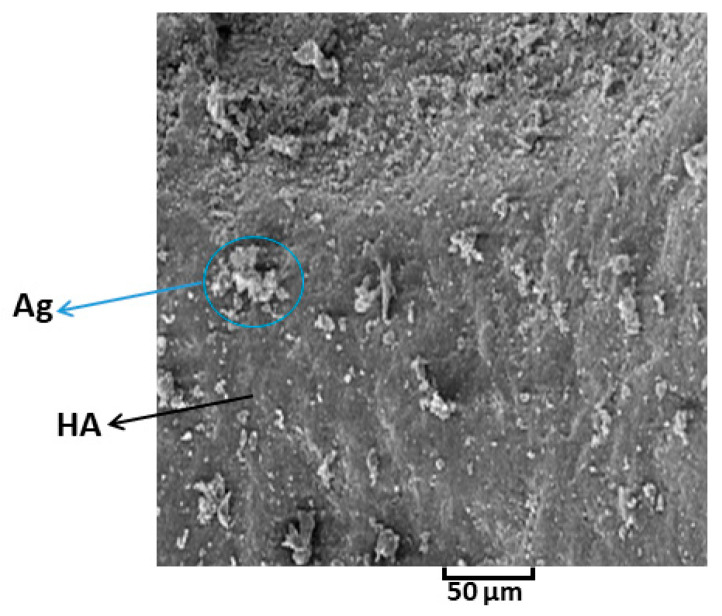
SEM image of Ag-doped HA powder.

The SEM image of Ag-doped HA powder is shown in Figure 9. The background of HA has a disciplined distribution and can contribute to the properties of bioactivity [57]. It is clear that the Ag is eager to cover the surface of HA, and the growth of Ag grains is depicted. Meanwhile, some aggregate-like particles begin to appear on the HA crystals, which can be related to Ag_3_PO_4_ [58]. The chosen sintering temperature is greatly affected by the prevention of decomposition of Ag_3_PO_4_ into metallic Ag through sintering process.

**Figure 10:**
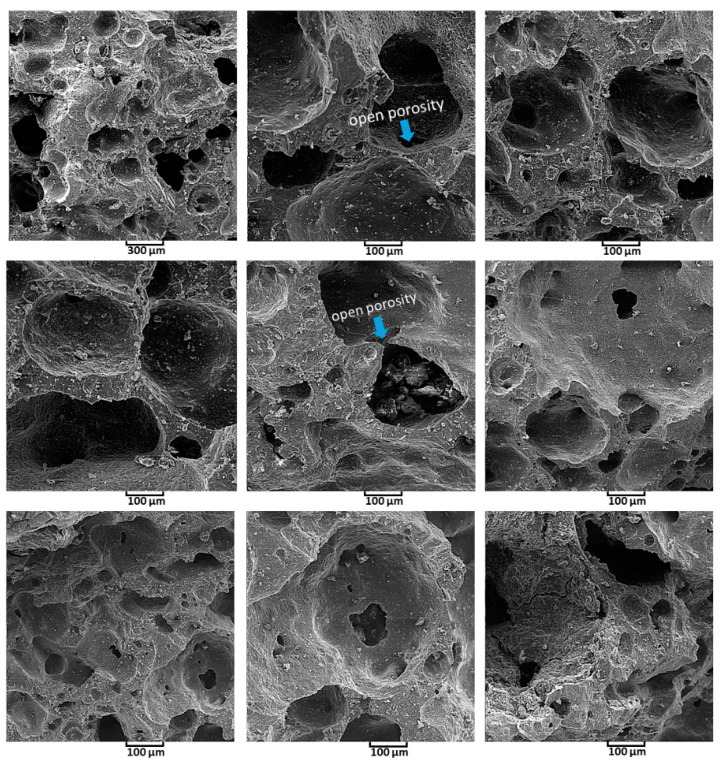
SEM images of Ag-doped HA+PVTMS scaffold.

SEM images of the Ag-doped HA+PVTMS scaffold are shown with different magnification in Figure 10. There is no existence of agglomeration in the scaffold. The effect of leakage (exit) of O, H and C in the rolled hair bond during heat treatment led to creation of porosities. The porosity size (>100 µm), the amount of porosity and the open porosity caused a high coefficient of bioactivity, because the porosities are the best place for bonding between calcium and phosphate groups. According to Figure 10, the uniformity of porosity is shown and the average porosity size is ~>200 µm, this value is suitable for the immigration of osteoblasts in porosities [59]. The best advantage of this study is related to create the porosity with high size without collapse of scaffold. Furthermore, according to studies, the minimum porosity size for significant bone growth is 75–100 µm with an optimal range from 100 to 135 µm [60,61]. Therefore, in this study, the porosity with a big size (higher than 200 µm) is produced. However, it is important to identify the upper limits of pore size, because the mechanical properties of the scaffolds of large pores may be affected by increasing the pore volume [62]. 

**Figure 11:**
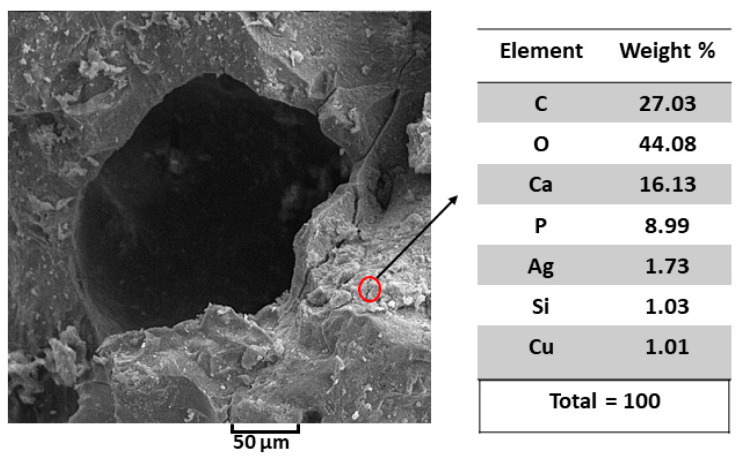
SEM EDAX data for Ag-doped HA+PVTMS scaffold.

The results of elemental analysis using EDAX stoichiometry of SEM are shown in Figure 11. According to the values of EDAX stoichiometry, there are no impurity elements except Cu, which serves as a reference in this analysis. The as-prepared Ag-doped HA+PVTMS scaffold showed a Ca/P ratio of 1.79, which is not far from the previously reported ratio of pure hydroxyapatite (1.67) [63]. The low Ag content in the scaffold is due to the AgNO_3_ content. The role of Ag is mainly related to the antibacterial properties, and this amount can support this feature. In this case, the substitution of Ag instead of Ca is very important, because it affects the chemical properties of hydroxyapatite, decreases the crystallinity and increases the solubility [64]. The EDAX analysis of the studied scaffold has confirmed the presence of all the constituent elements of Ag-doped HA+PVTMS.

### 3.5. Mechanical Properties

**Figure 12:**
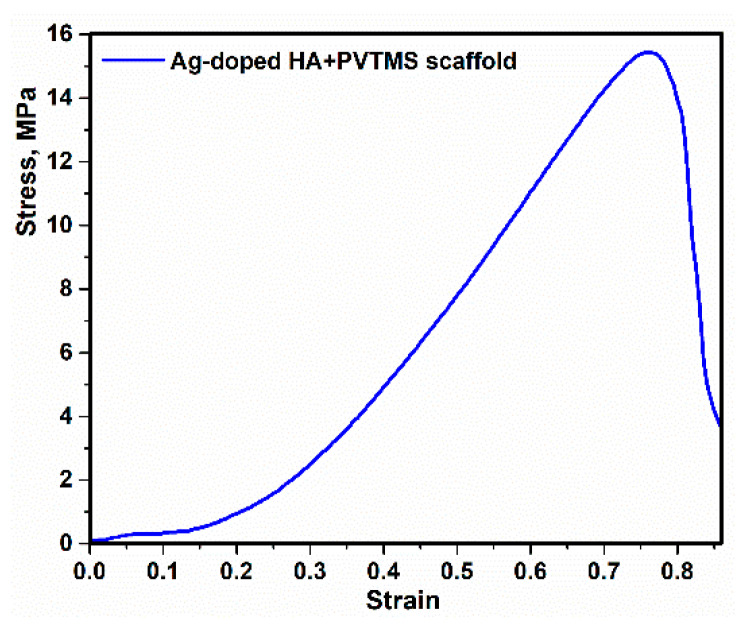
The curve of stress–strain compression of Ag-doped HA+PVTMS scaffold.

The stress–strain compression curve of the Ag-doped HA+PVTMS scaffold is shown in Figure 12. The maximum value of compressive strength is measured to be 15.71 MPa at a strain of ~0.77. According to Hooke’s law with the selection of the difference values of two points in the elastic region, the elastic coefficient is calculated ~3.86 MPa that this value is lower than the natural HA standard value, and it is associated with the porous scaffold and specifically the size of porosity is larger due to the focus of bioactivity in this study, [2,65]. The value of maximum compressive strength was not predicted according to the utilizing hair band as a mold. This value could be suitable because, (1) fabrication of scaffold through the using components such as Ag-doped HA (93 wt%)/PVTMS (5 wt%)/H_2_O (1 wt%)/CH_3_COOH (1 wt%) without using metals with high concentration. (2) In this case, the bioactivity characteristics and creating the large size of porosities were purpose and, considering the initial bioactivity of ingredients, use of high concentration metals such as Mg, Zn and Pt can improve the value of compressive strength and mechanical properties.

### 3.6. Investigation of Bioactivity

**Figure 13:**
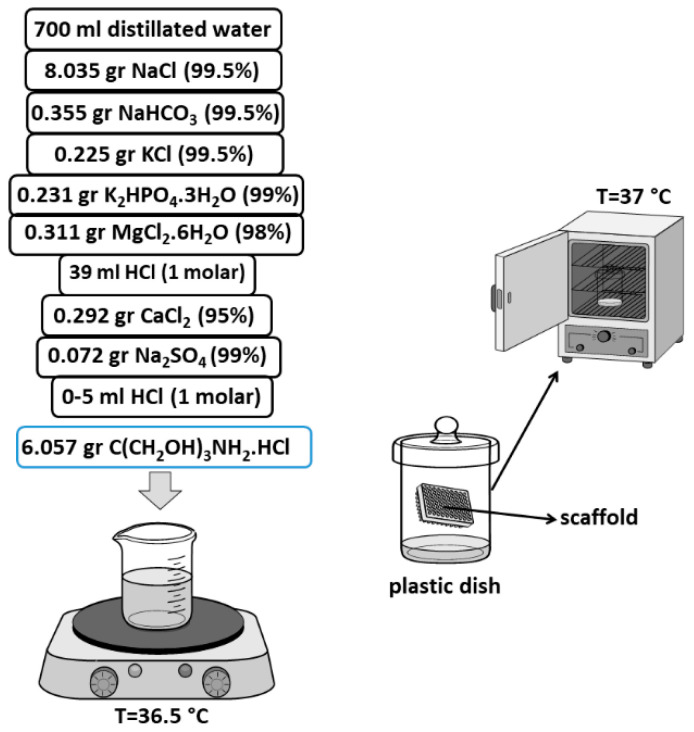
Synthesis of SBF [29], immersed scaffold in the SBF at 37 °C.

Scaffold was immersed in SBF (Figure 13) and kept in the oven at 37 °C (similar to body temperature). After 3, 5, 10 and 20 days, the scaffold was pulled out and washed with distillated water then, analysis such as XRD, FTIR, SEM and EDAX were performed. 

**Figure 14:**
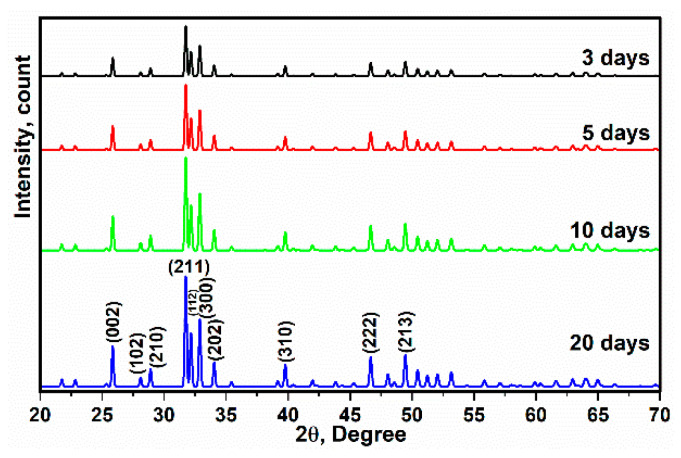
X-ray diffraction of immersed scaffold in SBF after 3, 5, 10 and 20 days.

**Figure 15:**
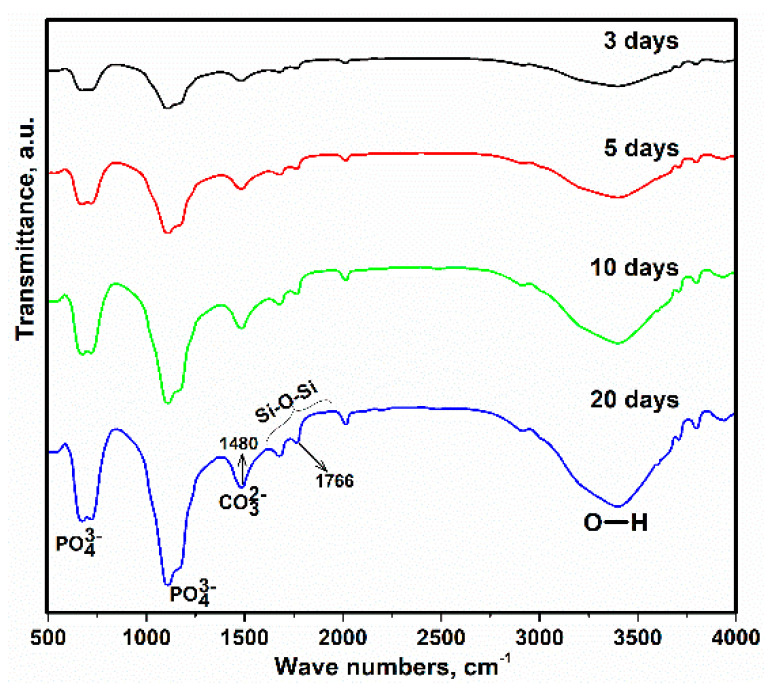
FTIR spectrum of immersed scaffold in SBF after 3, 5, 10 and 20 days.

**Figure 16:**
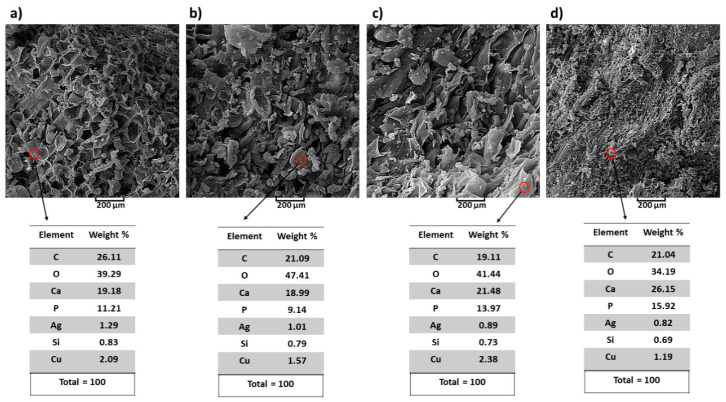
SEM EDAX data of immersed scaffold in SBF after (**a**) 3 days, (**b**) 5 days, (**c**) 10 days and (**d**) 20 days.

The X-ray diffraction and FTIR spectra, as well as the SEM images and EDAX data analysis of the scaffold after immersion in SBF, are shown in Figure 14, Figure 15 and Figure 16. According to the analysis, there are no additional ingredients or phases. In addition, it was interesting that imperfection of crystallization and replacements of ions were not observed corresponding to the sharp peaks (no amorphous) and the Ag has covered the surface of HA and impressive substitutions were not seen. It is clear that after 3 days the bonding between calcium and phosphate increased and the X-ray diffraction shows the HA pattern (based on HA matrix) (Figure 14). Furthermore, after 20 days, the intensity of the peaks increased, and based on the pattern, the strong diffraction peaks at 2ϴ values are attributed to the HA structure, whose hkl values of the exact natural HA peaks are assigned to 002, 102, 210, 211, 112, 300, 202, 310, 222 and 213, respectively [66]. The important result of this analysis is related to the positive presence of Ag and PVTMS in the scaffold and the large porosity, which help in the nucleation and growth of calcium and phosphorus ions from SBF [67], so that the rate of bioactivity increased with the appearance of HA after only 3 days of immersion. Therefore, the approach of choosing hair band as a porous form was very applicable.

In the FTIR spectrum (Figure 15), the weak peaks at ~1600–2000 cm^−1^ can be associated with the Si-O-Si groups originating from PVTMS or with the overlap of spectra attributed to the carbonate and silanol groups [68]. Moreover, the weak peak at 1766 cm^−1^ can be attributed to the potential occurrence of hydrogen bonding between the carbonyl, hydroxyl and phosphate groups. This evidence indicates the presence of a bioactive scaffold in the structure. The transmittance values increased over the days, suggesting more precipitation of HA on the surface of the porous scaffold. In addition, a broad peak in the range from 2800 to 3760 cm^−1^ is associated with the bonding between O and H, which occurs as a bending mode, and the reason for the presence of these peaks is related to the hydroxyl groups of the precursor and water. Additionally, the researchers reported that the peak at 1480 cm^−1^ belongs to C–O [69,70]. Thus, the FTIR spectrum agreed with the FTIR spectrum of natural HA [71].

According to the SEM images (Figure 16) of the pulled out scaffold (after immersion), synthesis of the viscose slurry as well as the existence of porosities and utilizing hair band as a novelty were useful and effective. The rate of precipitation of HA on the surface of scaffold was very high when the porosities were filled only after 3 days of immersion (Figure 16). In addition, with this idea (utilizing hair band as a mold) the diffusion of calcium and phosphor ions from SBF into the porosities was increased. Nevertheless, needle-shaped morphology (initial morphology) of HA [72] is converted to the sphere shapes. As mentioned in [73,74], these porosities are applicable according to the size of blood cells and requirements to the big porosities for siting the blood cells into the porosities and increasing the ratio of bioactivity. Taking into account the mechanical properties of porous scaffolds, whatever the amount and size of porosity are higher and bigger, respectively, the ratio of bioactivity will be increased [75]. According to Figure 16, the precipitation of HA increased over time of immersed scaffold. Taking into account the mechanism of precipitation and preparation of HA on the surface of scaffold, the existence of HCl in SBF is caused to the increasing of precipitation of HA due to the incrassating numbers of polar groups on the surface of scaffold and increasing of ratio of absorbance [76,77]. Figure 16 shows the result of EDAX of existence of HA on the surface during the 3, 5, 10 and 20 days. The ratio of Ca/P is reported 1.71, 2.07, 1.53 and 1.64 for immersed scaffold in SBF during the 3, 5, 10 and 20 days, respectively. It is clear that after 20 days of immersion, the ratio of Ca/P reached to 1.64 and this value is closer to the ratio of Ca/P = 1.67 from natural HA [78]. According to our study, the use of in vivo tests and the fabrication of this type of scaffold with the use of the bioactive metal part to increase the mechanical properties are unexplored aspects.

## 4. Conclusions

1. The synthesis of the composite consisted of Ag-doped HA through the utilizing mechanochemical process was successful. SPS process prevented temperature deviation and phase decomposition of composite, in addition, SPS was caused to fabricate uniformity of Ag-doped HA and as a result, the distribution of Ag in HA was desirable through using the SPS process.
2. The effect of Ag and PVTMS loading on the HA, was assessed. Ag provided antibacterial environment and PVTMS preven ted from cracking and shrinkage of scaffold and the free radicals of PVTMS structure provided flexibility of the scaffold through the bonding between C-C and siloxane (Si-O-Si), as well as PVTMS prevented to collapse via carbon chains during the heat treatment, because it is hydrophobic polymer due to the silane group.
3. The crystal size of Ag-doped HA was calculated 38 ± 2 nm and this value was corresponded with value extracted by TEM analysis (~less than 50). In addition, the a and c parameters (a = 9.59 Å, c = 6.86 Å) of the Ag-doped HA+PVTMS structure increased, which is due to the larger ionic radius of Ag compared to Ca and it is related to the proper bonding between Ag ions and the HA structure.
4. A new approach to fabricate porous scaffold through the utilizing hair band was carried out and as a result, the average porosity value was obtained at >200 µm, so this value was suitable for sitting blood cells in these porosities and the coefficient of bioactivity was enhanced.
5. For investigating in vitro bioactivity test, the SBF was successfully synthesized, and the scaffold was placed in the SBF for 3, 5, 10 and 20 days. As the result, according to the FTIR spectrum of immersed scaffold in SBF, the resulting bands were attributed to HA, with the most prominent bands related to the phosphate groups of the HA structure. In addition, the XRD, SEM and EDAX analysis of the immersed scaffold were studied and HA was nucleated and grown on the surface of the scaffold; as a result, the scaffold was bioactive.
6. The maximum value of compressive strength reached 15.71 MPa, and this value could be suitable according to the content of Ag and not using high amount of metals.

## Data Availability

Data sharing is not applicable.
